# Case Report: Eyelid reconstruction using intact fish skin xenograft following periorbital necrotizing fasciitis

**DOI:** 10.3389/fopht.2025.1589771

**Published:** 2025-05-22

**Authors:** Molly Shott, Diane Wang, John Nguyen

**Affiliations:** Department of Ophthalmology and Visual Sciences, West Virginia University, Morgantown, WV, United States

**Keywords:** fish skin graft, periorbital necrotizing fasciitis, eyelid reconstruction, xenograft, necrotizing fasciitis (NF)

## Abstract

Periorbital necrotizing fasciitis (NF) is a rare, vision-threatening soft tissue infection that requires rapid antimicrobial treatments to minimize devastating soft tissue destruction, and debridement of affected tissue results in extensive defects requiring various techniques of reconstruction. We herein describe a case of a 58-year-old male with periorbital NF who underwent eyelid repair with intact fish skin grafts. To our knowledge, this is the first case report of fish skin grafts being used in eyelid wound management after periorbital NF.

## Introduction

Necrotizing fasciitis (NF) is a rapidly progressive infection of soft tissue that spreads through fascial planes resulting in destruction of the surrounding tissue. If not recognized and treated quickly, the infection can spread systemically resulting in sepsis and possible death (1). Type 1 NF results from polymicrobial infection with anaerobes, gram negative bacilli, and enterococci. More commonly, type 2 NF is caused by beta-hemolytic strep species, particularly *Streptococcus pyogenes*, with or without a superimposed staphylococcal infection. Considering the virulence of these agents, proper treatment depends on initiation of broad spectrum intravenous antibiotics as well as prompt surgical debridement to remove affected tissue (2).

Though the mainstays of treatment remain the same for periorbital NF compared to NF in other areas of the body, extra considerations should be made considering the anatomic features of the eyelid and orbit. While NF of the orbital area is rare due to the area’s abundant blood supply and the orbicularis muscle serving as barrier to prevent spread deep into the orbit, when infection does occur, morbidity can be significant and range from cosmetic defects to loss of vision to death (3). Additionally, periorbital NF often occurs bilaterally due to the dermal attachments at the nasojugal and palpebromalar grooves and thickened dermis at the brow directing spread of infection medially across the nasal bridge. Due to the thin skin of the eyelids and lack of significant subcutaneous fat, necrosis occurs quickly. While this increases the urgency with which treatment must be sought, it also assures that the source of infection is apparent and unlikely to serve as a hidden nidus (4). Reconstruction of the eyelids after debridement needs to be done in a timely manner to protect the eye and orbit. However, it can be challenging and can often involve several stages to restore both form and function. Here the authors present a case of periorbital NF for which reconstruction was carried out using acellular fish skin graft (FSG).

## Case

**Figure 1:**
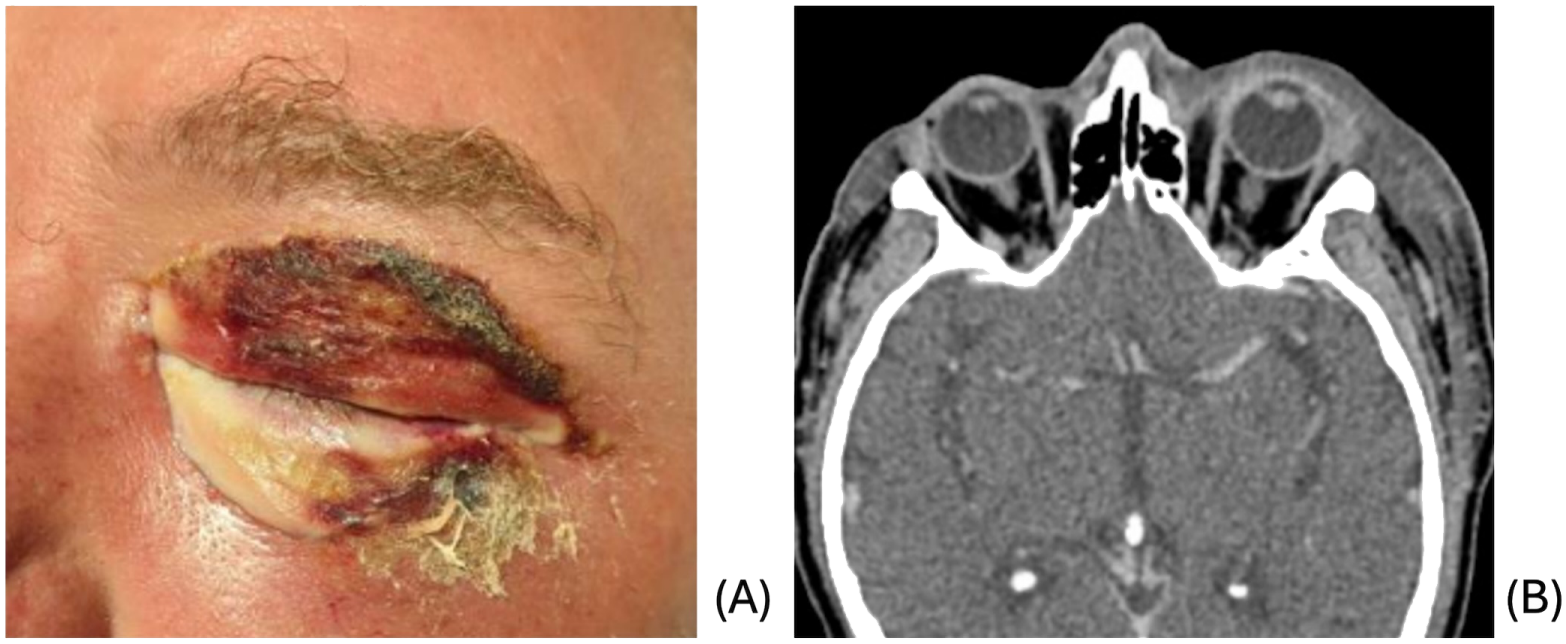
**(A)** Initial clinical presentation demonstrating periorbital edema and erythema with pale lower lid and scabbing of upper lid. **(B)** CT demonstrating extensive subcutaneous edema of the left face and preseptal cellulitis.

A 58-year-old male with a history of alcohol use disorder presented to the emergency department for 3 days of left facial swelling and pain. He reported doing yard work the day prior to symptom onset but denied trauma. He was tachycardic to 122 but afebrile and normotensive on presentation. Clinical examination revealed induration of left face from forehead to jaw with extensive left periorbital edema and erythema and pale appearance of the lower eyelids as well as scabbing of the upper eyelid (**Figure 1A**). The area was firm and mildly tender to palpation with no underlying crepitus or fluctuance. Initial workup was remarkable for WBC count of 16.5 and CRP of 343. He was admitted for concern of sepsis secondary to preseptal cellulitis.

CT of the face and neck revealed extensive subcutaneous edema of the left face with no focal fluid collection or soft tissue air, along with preseptal cellulitis, and clear sinuses (**Figure 1B**). Blood cultures grew *Streptococcus pyogenes* and *Serratia marcensens*. Ophthalmologic evaluation revealed normal vision, pupils, intraocular pressures, and fundus examination. The left upper and lower eyelids were mildly tender to palpation. The patient was started on ampicillin-sulbactam and linezolid due to concern for possible NF given clinical appearance and rapid progression.

**Figure 2:**
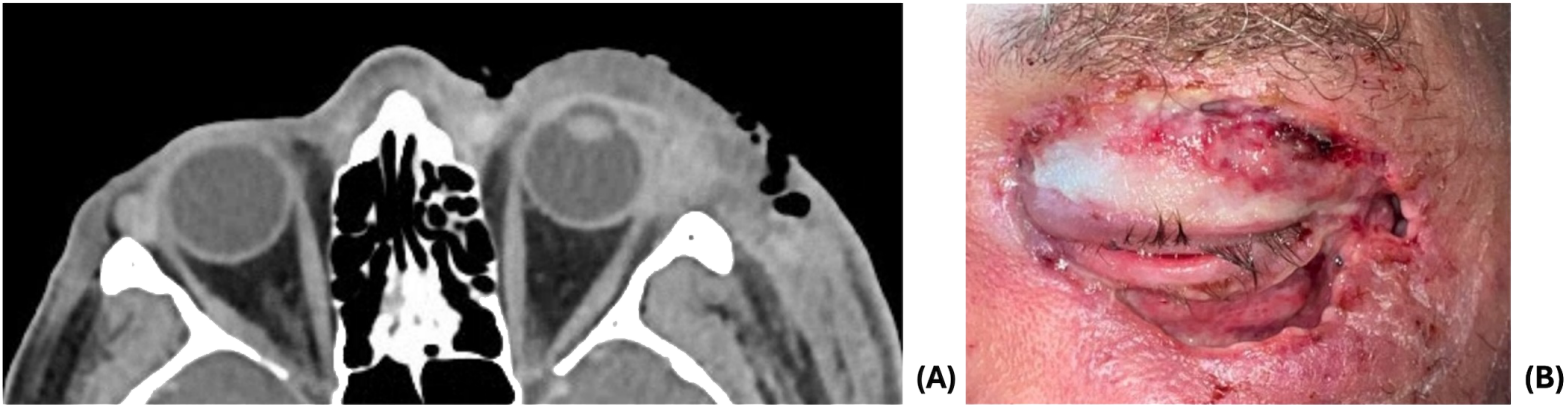
**(A)** CT demonstrating increased left periorbital soft tissue swelling extending to left-sided face and presence of gas within thickened skin and soft tissues. **(B)** 1 day post-debridement.

The patient’s periorbital edema and discoloration improved over the next 72 hours on broad spectrum antibiotics, and he was transitioned to oral amoxicillin-clavulanate. However, symptoms worsened, and antibiotics were re-escalated to vancomycin, cefepime, and metronidazole. His ocular exam remained stable despite worsening cellulitis. The decision was made to proceed with surgical debridement due to recurrence of edema of the eyelids and face with necrotic eyelids as well as worsening CT findings including the presence of gas within thickened skin and soft tissues (**Figure 2A**). The left upper and lower eyelids were debrided extensively (**Figure 2B**), with necrosis visualized past the orbital septum on the lower lids. The wounds were irrigated and packed with vancomycin. Cultures grew *Streptococcus pyogenes* and *Staphylococcus epidermidis*.

**Figure 3:**
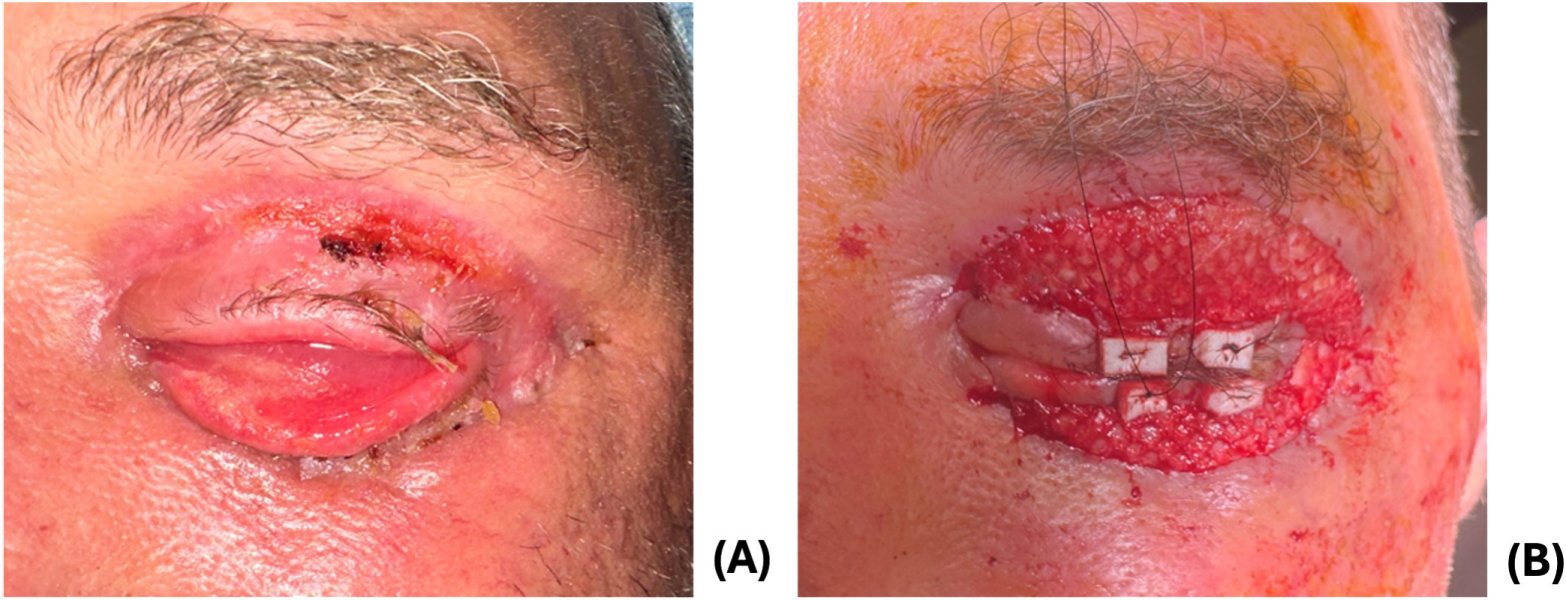
**(A)** 1 week post-debridement. **(B)** Appearance following Kerecis graft.

Following debridement, the patient’s cellulitis improved, and the patient was discharged on cefpodoxime, linezolid, and metronidazole. At the ophthalmology clinic 8 days later, his wounds were granulating and appeared well-perfused (**Figure 3A**). He had cicatricial changes that were worse on the lower eyelid compared to the upper eyelid. He underwent in-office release of cicatrix and repair with ​​FSG (Kerecis Omega3 Wound; Kerecis, Iceland) sutured using 5–0 monocryl and a temporary tarsorraphy (**Figure 3B**).

At two week follow up, the upper lid was well healed with improved lid closure, and he underwent further addition of FSG for the cicatricial left lower eyelid due to integration of the FSG. Adhesions between the septum and inferior orbital rim were lysed until the lower lid was freely mobile. The lower fornix was reformed using chromic sutures to invert the lower lid. Additional FSG was sutured over the final lower eyelid wound. Two weeks postoperatively, his lids were healing well with mild residual lower eyelid ectropion. The patient was instructed to apply antibiotic-steroid ointment to lower eyelid with upward massage to improve the eyelid position.

**Figure 4:**
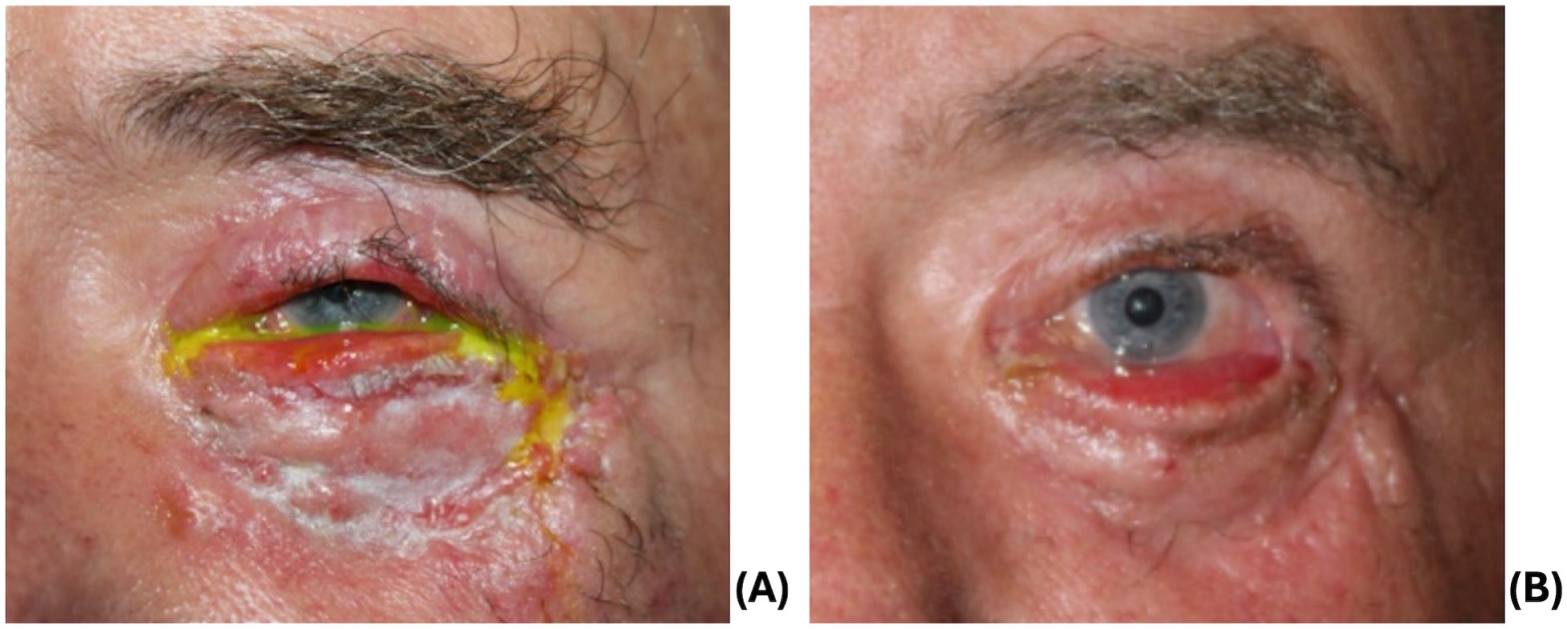
**(A)** 1 week post skin grafting. **(B)** 3 months post skin grafting.

One month later, additional repair was performed for recurrent septal scarring. He underwent a lateral tarsal strip and medial spindle, anterior lamellar full thickness preauricular skin graft (**Figure 4A**), and temporary tarsorrhaphy. At his follow up visit, he had mild ectropion of the lower lid (**Figure 4B**) and mild lagophthalmos without exposure keratopathy and a visual acuity of 20/25. Despite being offered additional reconstruction, he was subsequently lost to follow up.

## Discussion

Periorbital NF is a severe infection that requires prompt diagnosis and treatment to prevent devastating outcomes. A systematic review of 103 cases of periorbital NF completed in 2010 revealed a mortality rate of 14.2% with the greatest predictor of mortality being infection with beta-hemolytic *Streptococcus*, the most common etiologic agent of NF (1). Trauma to the area was identified in the majority of these cases, however no inciting cause could be identified for approximately one third of the patients. Unlike in other locations of the body, periorbital NF is not typically associated with chronic illness or advanced age (2). A review of 11 patient cases completed by Tambe et al. found that 5 of the 11 were either diabetic or on immunosuppressive therapy (5). Luksich et al. similarly reports diabetes as a risk factor as well as alcohol use disorder, a factor present in this reported patient (4).

The current standard of treatment involves a multidisciplinary approach consisting of broad spectrum intravenous antibiotics and surgical debridement. Dilute hypochlorous acid, typically in concentrations of 0.01-0.03%, directly neutralizes the exotoxins produced by Group A streptococcus and can be administered via direct irrigation during debridement, soaked in gauze for wound dressing, or instillation through a Penrose drain (6). Debridement of NF should be limited to subcutaneous tissue in order to preserve skin and orbicularis muscle for improved healing (2). Following proper infection control, reconstruction can begin. Reconstruction after debridement often poses a challenge and may require multiple stages to restore eyelid function and appearance. In this reported case, extensive debrided wounds on the upper and lower eyelids were repaired in several stages using FSG as a primary therapy for the upper eyelid and an intermediate step for the lower eyelid.

Previously reported techniques after periorbital NF typically consist of rotational skin flaps and grafts (5, 7–9). Dermal substitutes have also been reported in a small case series (10). In many cases reported in the literature, reconstruction required multiple surgeries due to wound contracture leading to cicatricial lid retraction, lagophthalmos, and corneal exposure. In an attempt to avoid these late complications, Wladis presented a modified technique for wound closure. In this technique, following debridement, the wound edges were reopposed using interrupted 6–0 sutures placed 4 mm apart. Iodine-soaked gauze was packed into the wound between each suture and changed every 48 hours. Direct closure of the wound was performed after 2–3 dressing changes when the tissue began to heal. Of the 7 patients who underwent this technique, only 2 experienced mild lagophthalmos of approximately 1 mm warranting skin grafting to the upper eyelid. The remaining 5 patients required no additional procedures and experienced no exposure keratopathy (11).

Recently, a novel approach to wound repair has been described using FSG, a xenograft made of acellular fish skin. Because the proteoglycans, glycoproteins, lipids, and structural proteins in the extracellular matrix of fish skin are compositionally similar to human skin, grafting promotes wound healing by providing structure for the growth of epidermal cells and vasculature. The high concentration of omega-3 fatty acids in fish skin also provides anti-inflammatory properties to the healing tissue (12). A 2023 report by Dueppers et al. presents 2 cases of leg ulcers resulting from surgical treatment of NF treated with intact fish skin grafts. The authors noted improved tissue granulation of the wounds as well as pain relief reported by the patients. Both were consequently able to undergo successful repair using autologous skin grafts (13). Posner et al. similarly found that FSG resulted in rapid healing and improved pain in a patient with full thickness forearm wounds secondary to debridement of NF (14). Additionally, a recent study introduced the efficacy of fish skin grafting for periocular Mohs defects in six patients with basal cell carcinomas and squamous cell carcinomas affecting the eyebrow, nasal wall, and eyelids. One patient required a secondary application of FSG due to incomplete wound healing. Another required three rounds of FSG due to a large Mohs defect and subsequently underwent full-thickness skin grafting for a mild lower punctal ectropion. All six patients healed well with cosmetically acceptable results (15). Fish skin grafting may also provide a cost-savings benefit in addition to its clinical utility. A 2023 study comparing the outcomes of diabetic foot ulcers treated with fish skin grafts versus those treated with collagen alginate therapy revealed 25.5% more wound healing and a $2,818 annual saving for those treated with fish skin (12).

Limitations to be considered in the use of FSG for wound healing include allergies or sensitivities to fish as well as the possibility of graft failure. Additionally, there is a risk of skin contraction requiring further reconstruction as seen in this case. Some cases may warrant multiple rounds of grafting to achieve optimal results. In this reported case, the patient underwent 3 rounds of eyelid reconstruction. Although his result was suboptimal, he had significantly improved eyelid position and had excellent vision compared to post-debridement. While a dermal replacement such as Integra is another option prior to skin grafting, its usage may have led to a similar result. Hopkins et al. reported a series of 5 patients with periorbital NF who underwent an average of 2.8 debridements; 4 of the 5 patients continued to have lagophthalmos and/or ectropion after reconstruction even after receiving Integra (16). Futures studies including more patients who have undergone periocular reconstruction using FSG after necrotizing fasciitis, as well as comparisons with other dermal substitutes, are warranted.

To the authors’ knowledge, this is the first case to report the use of FSG in the reconstruction of periocular wounds secondary to NF. The aggressive course of NF leads to tissue necrosis and deep open wounds after debridement. FSG is a useful adjunct to optimize the wound bed and reduce contracture prior to definitive skin grafting of the injured eyelid. The use of FSG is quick, can be performed in-office or at bedside, and multiple layers can be applied. Given that patients with NF are acutely ill and require long hospital stays, another advantage of using FSG is to temporize the wound and allow the patient to heal further prior to undergoing more invasive skin grafting or rotational flap procedures. Patient compliance and adherence to follow-up is critical given the multi-stage process of wound repair and reconstruction using this route. Though the patient in this reported case had a relatively short 3-month follow-up after his most recent reconstruction, there was significant progress in his lid position despite large and extensive wounds. This case demonstrates the potential benefits of using FSG in wounds after periorbital NF, in which eyelid reconstruction frequently poses a challenge.

## Data Availability

The original contributions presented in the study are included in the article/supplementary material. Further inquiries can be directed to the corresponding author.
